# Entomological impact and social participation in dengue control: a cluster randomized trial in Fortaleza, Brazil

**DOI:** 10.1093/trstmh/tru187

**Published:** 2015-01-19

**Authors:** Andrea Caprara, José Wellington De Oliveira Lima, Ana Carolina Rocha Peixoto, Cyntia Monteiro Vasconcelos Motta, Joana Mary Soares Nobre, Johannes Sommerfeld, Axel Kroeger

**Affiliations:** a University of Ceará State (UECE), Centro de Ciências da Saúde, Department of Public Health, UECE Av. Dr. Silas Munguba, 1700, Itaperi- Fortaleza, 60740-000, CE Brasil; b Special Programme for Research and Training in Tropical Diseases (TDR), World Health Organization, Geneva, Switzerland; c Liverpool School of Tropical Medicine, Liverpool, UK

**Keywords:** Brazil, Cluster randomized trial, Dengue, Intervention study, Social participation, Vector management

## Abstract

**Background:**

This study intended to implement a novel intervention strategy, in Brazil, using an ecohealth approach and analyse its effectiveness and costs in reducing *Aedes aegypti* vector density as well as its acceptance, feasibility and sustainability. The intervention was conducted from 2012 to 2013 in the municipality of Fortaleza, northeast Brazil.

**Methodology:**

A cluster randomized controlled trial was designed by comparing ten intervention clusters with ten control clusters where routine vector control activities were conducted. The intervention included: community workshops; community involvement in clean-up campaigns; covering the elevated containers and in-house rubbish disposal without larviciding; mobilization of schoolchildren and senior inhabitants; and distribution of information, education and communication (IEC) materials in the community.

**Results:**

Differences in terms of social participation, commitment and leadership were present in the clusters. The results showed the effectiveness of the intervention package in comparison with the routine control programme. Differences regarding the costs of the intervention were reasonable and could be adopted by public health services.

**Conclusions:**

Embedding social participation and environmental management for improved dengue vector control was feasible and significantly reduced vector densities. Such a participatory ecohealth approach offers a promising alternative to routine vector control measures.

## Introduction

Dengue is a serious infectious disease prevalent in tropical regions of Southeast Asia, the South Pacific, East Africa, the Caribbean and Latin America, putting about 2.5 billion people worldwide at risk.^1^ It is caused by an arbovirus of the *Flaviviridae* family with four sero-types: DENV 1, DENV 2, DENV 3 and DENV 4. The incidence and severity of this disease depends on multiple factors such as the social context and the biological characteristics of the virus, vector and host.^1–3^ The dispersion of dengue has social, biological, ecological, political and economic elements, which characterize it as a complex problem requiring a systemic approach for its control.^3^ Its high incidence has traditionally been related to climatic aspects, temperature, humidity, population density, and the availability of water containers for vector breeding.^4–6^

During the 1980s, the magnitude of the dengue problem in the Americas increased considerably.^7^ Brazil reported 78% of all cases and accounted for 61% of all cases reported to WHO in 2001–2005, placing it first in the international ranking of notified dengue cases^8^. Within the country, the northeastern region of Brazil reported the second highest number of dengue cases per year in 2008. Most importantly a particular hot spot was reported by Fortaleza with a total of 31 491 confirmed^9^ dengue cases during that period (57.6% of the 54 661 State confirmed cases).^10^

Fortaleza is vulnerable to infestation by *Aedes aegypti* due to its tropical climate and high demographic density. The rapid population growth (4.88%, from 2 452 185 in 2010 to 2 571 896 in 2014)^11^ is suggestive of disordered urbanization with inadequate sanitary conditions including a deterioration in the health infrastructure that creates favourable conditions, particularly for vector borne diseases such as dengue.^12^ The irregularity and, at times, lack of water supply leads people to store water in various containers such as water tanks, cisterns, barrels, drums, bowls, pots, water filters and others.^5,12^

According to the Municipal Plan for Prevention and Control of Dengue^13^ the municipality of Fortaleza currently has a total of 1338 professionals working on dengue control actions with an emphasis on mechanical, chemical and biological vector control. Despite these efforts, the years from 2008 to 2012 were considered as a period of major epidemics (21 935 confirmed cases in 154 municipalities and the capital with 8044 confirmed cases^13^).

It has been repeatedly suggested^12,14^ that dengue vector control requires interventions with intersectorial partnerships, the involvement of local communities and integrated vector management. The study described in this article was part of a multicentre research initiative carried out in five Latin American countries to test novel approaches to dengue vector control. The initiative was based on a research partnership between the Special Programme for Research and Training in Tropical Diseases (TDR), and the International Development Research Centre of Canada (IDRC) entitled ‘Towards improved Dengue and Chagas Disease Control through Innovative Ecosystem Management and Community-Directed Interventions: An Eco-Bio-Social Research Programme on Dengue and Chagas Disease in Latin America and the Caribbean’.

In the initial project phase (2010–2011) a situational analysis was conducted to characterize and map the urban ecosystem, particularly the vector ecology. The analysis of vector's ecological patterns showed that 43.6% of *Aedes* pupae (as a proxy for adult vectors^15^) were found in containers used to store water (mainly water tanks on the roof or the ground) and 56.4% were from small discarded containers, filled mainly by rain water, which were not used for storing water. This study intended to control both productive container types and discarded containers through an ecohealth approach (ecosystem) and analyze its effectiveness in reducing *Aedes aegypti* vector density.^16^

## Materials and methods

The city of Fortaleza, capital of Ceará State, is situated on the Atlantic coast of north-eastern Brazil, on a latitude 3**°** 43′ 02′ and a west longitude 38**°** 32′ 35″. The area has an average elevation of 16 m, a warm and sub-humid tropical climate, with average temperatures between 26 and 28**°**C. The rainy season is from January to May with an average annual rainfall of 1338 mm.

A cluster randomized controlled trial was designed comparing 10 randomly selected intervention clusters with 10 control clusters. A geographical grid sampling method using satellite images was employed to randomly select 10 grid cells using geographic information system (GIS) technology and the ArcView GIS^17^ that allows the visualisation and analysis of GIS information. A cluster of approximately 100 households was identified in each grid The 10 clusters were paired with 10 others (10 pairs) according to similar ecological and sociological parameters. Then the clusters in each pair were randomly assigned to either the intervention or control arm of the study.

During the pre-intervention dry season (June to December 2012), the total rainfall was 187.7 mm. The pre-intervention entomological survey was conducted in November and December 2012. The rainy season (January to May 2013), in which the intervention was developed, had a total rainfall of 427.7 mm. The intervention was developed from January to April 2013 and the entomological survey was carried out in May 2013 during the post-intervention period.

At the initial stage of the project, key individuals were identified in each cluster: community members (C), community leaders (L), professionals related to the municipal endemic diseases control program (E) and professionals working at the health centres (S). Subsequently the intervention was implemented in the following way: in all the intervention clusters, environmental management activities were organized targeting those water containers which were identified in the baseline survey as producing more than 70% of all *Aedes pupae* (as a proxy measure for adult densities). The intervention focused on different principles of the ecohealth approach such as sustainability, based on removing discarded small recipients, cleaning backyard areas and covering large water containers without the utilization of larvicides or insecticides. Another important ecohealth principle,^16^ applied in the research, was community participation: community groups (elders' groups, students, community members) were mobilized and empowered at different levels (see below).

### Intervention methods

The intervention targeted productive container types, mainly small discarded and unused water containers stored in backyards and large water tanks as determined by the situational analysis. The strategy included: establishing partnerships, meeting with intersectorial groups to explain the objectives and procedures of the activities in the homes; requesting the Regional Secretariat for a truck for waste collection; organizing social mobilization through groups formed by National Health Service professionals, educators and Endemic Disease Agents (EDAs) who made home visits, delivered garbage bags, informed the community about the date on which the garbage truck was going to collect the trash and provided general health information.

Particular activities were the following:

#### Community workshops

The aim was to empower the community and establish a co-management group, stressing the individual and collective responsibility for dengue prevention, so that they could act as multipliers in the community. To this end, invitations were sent to individuals (professionals from the Family Health Strategy and the Department for Endemic Disease Control, Social Educators, community leaders, and community members) to participate in workshops, in which the group discussed the results of the situational analysis and planned actions. At each meeting the stakeholders themselves planned, monitored and participated in new actions in accordance with the needs of each locality.

#### Involving the community directly

The community helped to organize the meetings, was actively involved during clean-up campaigns, and took responsibility for cleaning the surrounding areas of public spaces.

#### Mobilizing schoolchildren and the elderly regarding dengue prevention

Partnerships were developed with schools on dengue control and solid waste management under the assumption that these spaces naturally inspire the adherence of social actors and enable the understanding that health is the responsibility of different sectors of society.^18^ Many schools have dedicated areas where the elderly can meet for leisure, gymnastics and other social and cultural activities; this facilitated the organization of activities with elderly people who actively participated in the distribution of information materials and helped with the cleaning of backyards.

#### Distributing information, education and communication (IEC) materials in the community

All the households in the intervention clusters received an educational calendar made by the research group together with the health authorities, which was intended to act as a reminder throughout the year of the actions that should be carried out by the residents in their homes to support dengue prevention.

### Data collection

Multidisciplinary research methods led to a triangulation of evidence. Standard entomological survey methodologies resulted in quantitative evidence on vector densities. Participatory research facilitated the design and conduct of community-based interventions. Social and anthropological field research (key informant interviews and participant observations) led to mainly qualitative data and evidence about social participation and community empowerment in the intervention clusters.

For the cost analysis, data collection tools were developed to measure resource consumption in physical units and gather data to value each resource item (unit cost). The local team in charge of the research project collected the information based on direct observation, field reports, expenditure reports and interviews. Comparable information was requested from the agencies in charge of the routine activities, however, obtaining such information proved challenging.

During the intervention period, the researchers were able to analyse the process of empowerment-collaboration-mobilization by means of indicators applied by Draper K, Hewitt G and Rifkin S.^19^ The framework considers five key indicators for community participation: leadership, planning and management, involvement of women, external support and monitoring and evaluation. The scores are marked on a scale of 1–5 for each question*.* The researchers applied the scores that mapped the respective intensity of community participation. Subsequently these indicators permitted the construction of spidergrams.

The entomological surveys, conducted by 10 trained professionals of the vector control services included the following: during the dry season a pre-intervention survey was carried out in all 20 study clusters; 4 weeks after completing the intervention, during the rainy season, the post-intervention survey was conducted using the same methodology. The change of ‘larval indices’ as a proxy measure of adult vector densities^15^ from before (dry season) to after (rainy season) the intervention were assessed.

### Data analysis

Quantitative data were entered and verified into database by using Microsoft Office Excel software (Microsoft Corporation, Redmond, WA, USA) and analysed with statistic software Stata (StataCorp, College Station, TX, USA). The variation of the house index (HI), the container index (CI), Breteau index (BI) and pupae per person (PPI) (i.e., larval indices) from the dry season (before intervention) to the rainy season (after the intervention) was assessed by means of linear mixed models.

Qualitative data were recorded, transcribed and transferred to a central database using NVivo software package (QRS International Pty Ltd., Doncaster, Victoria, Australia). Each text transcript was then coded and coding categories were derived through identifying common responses in the clusters.

Cost items were classified according to the resources consumed (personnel, consumables, transport operating costs, and other costs incurred in meetings with the community) and then descriptively analysed and aggregated to calculate total costs and costs per house reached. To standardize reporting, costs collected in local currency were converted to US dollars (US$) using average exchange rates for the year of implementation of the interventions (the interventions started in 2013).

## Results

### Achievements regarding reduction of vector breeding places

**Figure 1.**
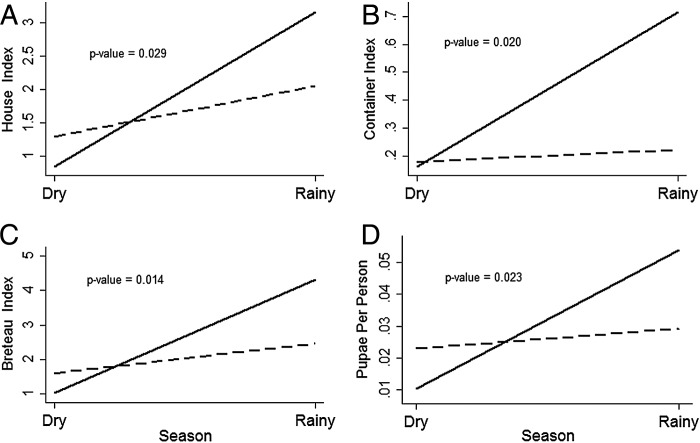
*Aedes aegypti* infestation, before (dry season) and after (rainy season) the intervention. —— Control area; ––– Intervention area.

At the end of the intervention all of the large tanks in the intervention clusters were covered with the help of the EDAs. This was achieved through education activities with the intersectoral group (family health strategy professionals, social mobilizers, EDAs) allowing residents to maintain the covers of the large tanks in perfect condition (see Table 1) which helped to reduce the CI and PPI as shown in Figure 1. There was an important reduction in small discarded water containers in the intervention clusters (100% elimination in all visited houses) while in control clusters water containers were treated according to the National Control Programme (using insecticide when containers hold more than 200 litres and emptying the smaller ones).

**Table 1.** Water tanks covered by the eco-bio-social integrated intervention to control *Aedes aegypti* in Fortaleza, Ceara state, Brazil, 2013

Cluster	Neighborhood	Total water tanks	Covered water tanks	Tanks covered by the eco-bio-social project
1	Messejana	43	42	1
2	Vila Ellery	52	29	23
3	Quintino Cunha	65	61	4
4	Pici	89	83	6
5	Passaré	53	40	13
6	Parreão	84	82	2
7	Centro	102	84	18
8	Granja Lisboa	40	38	2
9	José Walter	68	53	15
10	Papicu	32	23	9
Total		628	535	93

### Economic considerations

The distribution of costs for the interventions in households showed that staff costs (11 EDAs and 1 field coordinator during 7 months valued at US$185 and US$277 per month respectively) was the most important component accounting for 85.8% of total costs. It is noteworthy that, the intervention was delivered by staff routinely working in the vector control programme and required a similar team and staff-time as the routine activities. There were also expenditures for consumables (garbage bags, calendars and prints; lids and covers for the large water tanks) and workshops and community meetings (Table 2). The total costs of the intervention was US$18.89 per house, and the costs related to the ecohealth intervention were around US$2.23 per house

**Table 2.** Cost (US$) of materials and services consumed by the eco-bio-social integrated intervention of control *Aedes aegypti* in Fortaleza, Ceara state, Brazil, 2013

Clusters	Garbage bags	Tanks covered	Calendars	Leaflets	Meetings	Total for cluster
Parreão	20.79	33.73	85.11	55.81	28.13	223.58
Messejana	20.79	7.49	85.11	55.81	42.20	211.41
Quintino Cunha	20.79	74.97	85.11	55.81	37.51	274.20
José Walter	20.79	97.46	85.11	55.81	37.51	296.69
Passaré	20.79	18.74	85.11	55.81	18.75	199.21
Centro	20.79	0	85.11	55.81	0	161.71
Granja Lisboa	20.79	0	85.11	55.81	0	161.71
Pici	20.79	7.49	85.11	55.81	0	169.21
Vila Ellery	20.79	0	85.11	55.81	0	161.71
Papicu	20.79	11.25	85.11	55.81	0	172.97
Total for consumables	207.79	251.16	851.16	558.13	0	2032.50

### Impact on vector densities

Two cross-sectional larvae and pupae surveys were carried out between January and June 2013 in both control and intervention areas. A total of 2411 places were visited in both dry and rainy seasons (2353 households and 58 public spaces) (Table 3). Before and after the intervention strong differences were identified between the intervention and control areas (Table 4): overall, the HI, CI, BI and PPI increased, as expected, from the dry season (before intervention) to the rainy season (after the intervention), but the increase was significantly higher in the control area (p-values: HI=0.029 CI:=0.020, BI=0.014, PPI=0.023) demonstrating the protective efficacy of the intervention (Figure 1).

**Table 3.** Characteristics of the study area before the eco-bio-social integrated intervention to control *Aedes aegypti* in Fortaleza, Ceara state, Brazil

Characteristics	Area	
	Control	Intervention
Number of households	1580	1689
Number of inhabitants	4058	4123
Number of large containers^a^	776	1032
Number of small containers^b^	2823	3519
Number of potential containers	1408	1585
Number of discarded materials^c^	4488	16 223

^a^ Volume ≥200 litres (e.g., water tanks, tanks, drums, cacimbas, pools).

^b^ Volume <200 litres (e.g., buckets, basins, pots, filters).

^c^ Discarded materials, recyclable garbage.

**Table 4.** Entomological indices in the intervention and control areas, in the dry and rainy season, achieved by the eco-bio-social integrated intervention to control *Aedes aegypti* in Fortaleza, Ceara state, Brazil, 2013

Indicators	Dry season		Rainy season		p-value
	Control	Intervention	Control	Intervention	
House index (%)	0.8383	1.2944	3.1664	2.0497	0.029
Container index	0.1625	0.1799	0.7157	0.2228	0.020
Breteau index	1.0278	1.5991	4.3158	2.4646	0.014
Pupae per person	0.0104	0.0229	0.0539	0.0292	0.023

### Empowerment of the communities

**Figure 2.**
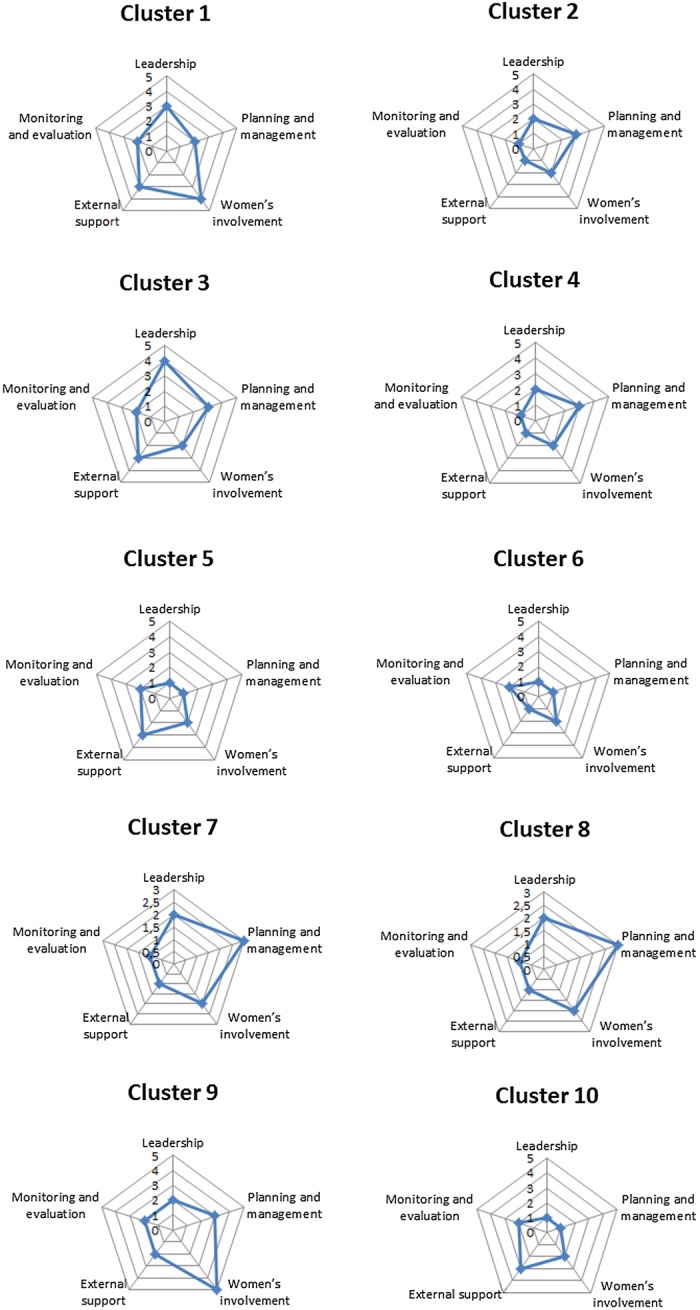
Spidergrams assessing five indicators of community participation. This figure is available in black and white in print and in color at Transactions online.

Empowerment is related to the process of giving groups or communities autonomy and a progressive and self-sustained improvement of their lives.^20^ The level of social participation was analysed by constructing spidergrams (Figure 2). The indicators helped in the analysis of the intervention of each locality. For example, in cluster 3 the spider graph illustrated high levels of leadership. This was due to the specific socio-cultural context of that community where historically the community organization was strong to deal with local issues. On the other hand, cluster 6 had the lowest level of leadership, which reflects the dynamics of a community that does not have a community organization. Cluster 9 showed high gender initiative and frequently mentioned in their discussions the crucial role of the women living in the neighbourhood in actively preventing transmission of dengue through neighbourhood mobilization.

Differences in terms of social participation, commitment, and leadership capacity were observed and documented in the clusters. Some clusters (e.g., 3 and 9) managed to organize the garbage collection with their respective Regional Secretariats and communities while others (e.g., 6 and 5) were more passive and achieved only minimal collaboration. In cluster 6 the community leadership was weak or almost non-existent^21^ as demonstrated in the speech of this health educator (cluster 6):

I felt the difficulty of the population involvement since the beginning. People are separate, they don't unite, I see people as selfish, I might be extreme but I see each one in his or her corner. I think your work is very interesting, but I see their lack of interest …

In clusters 5 and 6 these factors hindered the presence of social actors in the meetings, showing a moderate participation of women and a weak participation of health professionals. In other clusters (particularly 1 and 9) female participation was strong to very strong underlining their key role in the process of controlling dengue, because most of the time they are in charge of all the household activities.

In clusters 2, 4, 9 it was observed that the community leadership, external support, monitoring and evaluation scored 1. However, the presence of women scored widely from 4 to 5. In clusters 5 and 10, community leadership, planning and management, monitoring and evaluation scored poorly, but external support had a strong score, denoting a contradiction regarding the scenario when taking into consideration the absence of health and education professionals from the meetings in this cluster.

For the yard clean-up actions, individuals from other sectors assisted to collect the small discarded containers and unused materials. The EDAs seemed well motivated; the residents took their brooms and garbage bags and gathered the materials. At the end of the action, a community member said that the collaborative action was ‘a success’ (cluster 3). The company hired by the town hall for waste collection picked up the garbage which had been collected by the household members; they also helped to clean the public spaces in the clusters, removing rubble, disposable materials, and street garbage. Comments like this were frequent:

*I thought it was magnificent. It was organized as a social action, but it's always a big challenge. We can't always do this activity, but we know that there are many yards with buckets, bottles and other types of trash. It's a big challenge, because if you do it today and don't do it again it's complicated. But the idea remains of looking at the yard and recognizing breeding grounds for the vector (cluster 3)*.

This comment illustrates that cleaning backyards as a social event results not only in the elimination of breeding sites for the vector, but also as a form of creating awareness and motivation for the continuing care of the yard. One of the community leaders said:

There's a man who has a huge yard in his house, in the past we would get together and clean the yard for him. Then I said ‘No, we're wrong’. We have to teach because it's him who has to clean it. I can't leave my house and do it for him (cluster 3).

With the intervention, community participation was strengthened and elderly people and schools planned continuous actions together with the municipal workers.

## Discussion

Current dengue control is a complex activity, in view of the diverse factors external to the health sector, which are important determinants in the maintenance and dispersal of both the disease and its vector. Among these factors, one can highlight the emergence of urban settlements, inadequate housing conditions, irregularity in water supply, improper disposal of waste, the growing movement of people and cargo between countries and climate change caused by global warming.^16^

The study results showed the effectiveness of the ecohealth programme in terms of a significant reduction of the dengue vector population through targeted interventions in the most productive container types. The project also achieved an increase in peoples knowledge of dengue and willingness to participate in preventive actions. The intervention strategy was based on community participation and a partnership approach with public control services.

During the implementation process it was noted that the social participation of subjects and groups was heterogeneous and shaped by historical and actual community dynamics. Social participation was fragile in locations with nonexistent community organizations or in neighbourhoods with either a history of violence or very well off and privileged groups. The broader view of the determinants of dengue vector breeding served as a directing axis for designing and implementing the dengue control programme in intervention clusters.

Worldwide, one the most challenging approaches in achieving a successful outcome of dengue vector management, is related to the role of communities in eliminating domestic breeding sites.^22^ Community involvement is seen as essential in the control of endemic diseases, especially in the case of dengue, as the insect vector is closely associated with the lifestyle and housing in urban areas. Social participation may also have limitations, because health actions do not always occur in an orderly and continuous manner from an operational, political or institutional point of view.^18^ In our project areas, most of the clusters had a very weak community structure, which interferes with the positioning of people in relation to both individual and collective health care. However, when informing the population about the entomological goal of reducing potential vector breeding places to zero, the first step has been completed. But the next step is equally or even more important: to achieve changes in meaning and practices among the population.^20^

It must be emphasized that *Aedes aegypti* control requires the coordinated involvement of various sectors. These sectors include education, sanitation and street cleaning, culture, tourism, transport, construction and public safety; also partners from the private sector and organized society have to be approached and included.^21,23^ Our work in schools generated an environment for achieving significant changes in the health/illness, school/community, and educator/learner relationships.

### Limitations of the study

The analysis of community participation was based on Draper et al.^18^ The indicator's framework for community participation revealed differences among clusters but with some general tendencies: external support as well as monitoring and evaluation were weak in all the study clusters, suggesting a difficulty in dealing with intersectoriality in a ‘natural’ unforced manner, especially when led by the health sector. This is portrayed in the account of Lima and Vilasbôas^23^: ‘intersectoriality was restricted to a rhetorical level. The implementation of intersectorial actions remains a challenge to be overcome’.

Based on our results, in two mega-cities of the country. the Brazilian government has decided to implement the ecohealth approach described herein, accompanied by a monitoring programme to measure the impact on dengue incidence and also to assess the additional costs for the programme.

### Conclusions

Embedding social participation and environmental management for improved dengue vector control was feasible and significantly reduced vector densities. Such a participatory ecohealth approach offers a promising alternative to routine vector control measures carried out by services, often based solely on larviciding or space spraying and without social participation.

## References

[1] WHO (2009). Dengue: Guidelines for Diagnosis, Treatment, Prevention and Control.

[2] Gubler DJ (2002). The global emergence/resurgence of arboviral disease as public health problems. Arch Med Res.

[3] Santos SL, Augusto LGS (2011). Multidimensional model for dengue control: a proposal based on social reproduction and risk situations [in Portuguese]. Physis.

[4] Guzman MG, Kouri G (2008). Dengue haemorrhagic fever integral hypothesis: confirming observations, 1987–2007. Trans R Soc Trop Med Hyg.

[5] Teixeira MG, Costa MCN, Barreto F, Barreto ML (2009). Dengue: twenty-five years since reemergence in Brazil. Cad Saúde Pública.

[6] Wilder-Smith A, Ooi EE, Vasudevan SG, Gubler DJ (2010). Update on dengue: epidemiology, virus evolution, antiviral drugs, and vaccine development. Curr Infect Dis Rep.

[7] Timerman A, Nunes EP, Neto-Andrade JL (2009). First panel on dengue fever update [in Portuguese]. Rev Panam Infectol.

[8] World Health Organization Global Health Atlas. http://www.who.int/globalatlas/DataQuery/default.asp.

[9] Health Secretary of Fortaleza – SIMDA Dengue: cases by district of residence according to the onset of symptoms month, Fortaleza, 2008 [in Portuguese]. http://tc1.sms.fortaleza.ce.gov.br/simda/dengue/mes?ano=2008&modo=bairro&classifinold=1&criterio=&evolucao=&regional=.

[10] Ministry of Health Brazil – Health Data. Dengue Cases in Brasil. http://portalsaude.saude.gov.br/images/pdf/2014/julho/31/Dengue-classica-at---2013.pdf.

[11] IBGE (2010). Population Census 2010 [in Portuguese]. http://www.ibge.gov.br/home/estatistica/populacao/censo2010/default.shtm.

[12] Lópes TMT, Cordero JLG, Estrada JGS (2012). Cultural dimensions of dengue that help or hinder its prevention in Mexico [in Spanish]. Rev Panam Salud Publica.

[13] Health Secretary of Ceara State (2013). Bulletins [in Portuguese]. http://www.saude.ce.gov.br/index.php/boletins.

[14] Arunachalam N, Tyagi BK, Samuel M (2012). Community-based control of *Aedes aegypti* by adoption of eco-health methods in Chennai City, India. Pathog Glob Health.

[15] Focks DA (2003). A Review of Entomological Sampling Methods and Indicators for Dengue Vectors (TDR/IDE/Den/03.1). http://whqlibdoc.who.int/hq/2003/TDR_IDE_DEN_03.1.pdf.

[16] Bazzani R, Wiese M, Charron D (2012). Introduction. Ecohealth Research in Practice.

[18] Gohn MG (2004). Empowerment and community participation in social policies [in Portuguese]. Saude Soc.

[19] Draper K, Hewitt G, Rifkin S (2010). Chasing the dragon: developing indicators for the assessment of community participation in health programmes. Soc Sci Med.

[20] Streck DR, Streck D, Ghihhi G, Silveira FT, Pitano SC (2010). Popular Education and Participatory Research: The Construction of a Method [in Portuguese]. Readings of Paulo Freire: Contributions to Contemporary Educational Debate.

[21] Brasil (2009). Ministério da Saúde.

[22] Brassolatti RC (2002). Andrade CFS Evaluation of an educative intervention to prevent dengue [in Portuguese]. Ciênc Saúde Coletiva.

[23] Lima EC, Vilasbôas ALQ (2011). Inter-sector social mobilization for dengue controlin Bahia State, Brazil [in Portuguese]. Cad Saúde Pública.

